# Endovascular treatment for cerebral venous thrombosis: a multicenter study in China

**DOI:** 10.1186/s40779-025-00605-3

**Published:** 2025-04-08

**Authors:** He-Tao Bian, Xia Wang, Gui-You Liu, Chen Zhou, Ran Meng, Lan Liu, Jian-Gang Duan, Feng Yan, Chuan-Hui Li, Min Li, Wen Hui, Xu-Xiang Zhang, Dong Zhao, Ya-Peng Li, Qi Fang, De-Zhi Kang, Hong-Liang Zeng, Zhi-Jian Liang, Zheng-Hao Shi, Wei Yue, Qin-Jian Sun, Gui-Sheng Chen, Jian-Long Song, Zhong-Rui Yan, Qiu-Hong Ji, Kai-Jie Wang, Lu-Sha Tong, Xiao Hu, Wen-Feng Cao, Wei Yan, Rui-Jiang Gao, Qi Li, Jian-Yi Wang, Yi Liu, Bao-Jun Wang, Xiao-Hua Wang, Sheng-Tao Yao, Ye Lang, Hai-Peng Li, Craig S. Anderson, Xun-Ming Ji, on behalf of the RETAIN-CH Investigators and Coordinators

**Affiliations:** 1 Beijing Institute of Brain Disorders, Capital Medical University, Beijing, 100069 China; 2 The George Institute for Global Health, University of New South Wales, Sydney, 2000 Australia; 3 Department of Neurology, Xuanwu Hospital Capital Medical University, Beijing, 100053 China; 4 School of Statistics, University of Minnesota at Twin Cities, Minneapolis, MN 55455 USA; 5 Department of Emergency, Xuanwu Hospital of Capital Medical University, Beijing, 100053 China; 6 Department of Neurosurgery, Xuanwu Hospital of Capital Medical University, Beijing, 100053 China; 7 Department of Neurology and Stroke Center, Xuanwu Hospital of Capital Medical University, Beijing, 100053 China; 8 Department of Science and Technology, West China Hospital of Sichuan University, Chengdu, 610041 China; 9 Department of Ophthalmology, Xuanwu Hospital, Capital Medical University, Beijing, 100053 China; 10 Department of Center for Clinical and Epidemiologic Research, Beijing Anzhen Hospital, Capital Medical University, Beijing, 100013 China; 11 Department of Neurology, the First Affiliated Hospital of Zhengzhou University, Zhengzhou, 450002 China; 12 Department of Neurology, the First Affiliated Hospital of Soochow University, Suzhou, 215006 Jiangsu China; 13 Department of Neurosurgery, the First Affiliated Hospital of Fujian Medical University, Fuzhou, 350005 China; 14 Department of Neurology, Ganzhou People’s Hospital, Ganzhou, 341000 Jiangxi China; 15 Department of Neurology, the First Affiliated Hospital of Guangxi Medical University, Xining, 530021 China; 16 Department of Neurology, the First Affiliated Hospital of Wenzhou Medical University, Wenzhou, 325000 Zhejiang China; 17 Department of Neurology, Tianjin Huanhu Hospital, Tianjin, 300350 China; 18 Department of Neurology, Shandong Provincial Hospital Affiliated to Shandong First Medical University, Jinan, 250021 China; 19 Department of Neurology, General Hospital of Ningxia Medical University, Yinchuan, 750001 China; 20 Department of Neurology, the First Affiliated Hospital of USTC, Hefei, 230001 China; 21 Department of Neurology, Jining First People’s Hospital, Jining, 272113 Shandong China; 22 Department of Neurology, Affiliated Hospital of Nantong University, Nantong, 226001 Jiangsu China; 23 Department of Neurology, Tangshan Gongren Hospital, Tangshan, 063099 Hebei China; 24 Department of Neurology, the Second Affiliated Hospital of Zhejiang University School of Medicine, Hangzhou, 310009 China; 25 Department of Neurology, Guizhou Provincial People’s Hospital, Guiyang, 550002 China; 26 Department of Neurology, Jiangxi Provincial People’s Hospital, Nanchang, 330006 China; 27 Department of Neurology, the First People’s Hospital of Kashi, Kashi, 84000 Xinjiang China; 28 Department of Neurology, Inner Mongolia People’s Hospital, Hohhot, 010010 China; 29 Department of Neurology, the Second Affiliated Hospital of Anhui Medical University, Hefei, 230601 China; 30 Department of Neurology, the First Affiliated Hospital of Xi’an Jiaotong University, Xi’an, 710061 China; 31 Department of Neurology, Shanxi Provincial People’s Hospital, Taiyuan, 030012 China; 32 Department of Neurology, Baotou Central Hospital Inner Mongolia, Baotou, 014040 Inner Mongolia Autonomous Region China; 33 Department of Neurology, Qujing No. 1 Hospital, Qujing, 655000 Yunnan China; 34 Department of Neurology, Affiliated Hospital of Zunyi Medical University, Zunyi, 563099 Guizhou China; 35 Department of Neurology, Shengli Oilfield Central Hospital, Dongying, 257100 Shandong China; 36 Department of Neurology, the First People’s Hospital of Chenzhou, Chenzhou, 424300 Hunan China; 37 Institute of Science and Technology for Brain-inspired Intelligence, Fudan University, Shanghai, 200433 China

**Keywords:** Cerebral venous thrombosis (CVT), Endovascular treatment (EVT), Standard care, Efficacy, Safety

## Abstract

**Background:**

Endovascular treatment (EVT) is gaining popularity for the management of severe forms of cerebral venous thrombosis (CVT), but the evidence supporting its efficacy and safety is limited.

**Methods:**

This multicenter study included patients with CVT admitted to 104 hospitals in 31 provinces/cities in China between January 2018 and June 2022. Propensity score weighting models were used to adjust baseline confounding variables to determine the association of EVT on the primary outcome of good functional status, defined as score 0 − 1 on the modified Rankin Scale after hospital discharge.

**Results:**

Of 3063 patients identified through hospital records searches, 2774 adults [age (42 ± 15.8) years, female 50.3%] fulfilled eligibility criteria and agreed to be included, of whom 449 (16.2%) received EVT and 2325 (83.8%) received standard care. There was no significant difference between the EVT group and the standard care group in terms of the possibility of good functional recovery [weighted risk ratio = 1.00, 95% confidence interval (CI) 0.96 − 1.03]. Similarly, there was no difference in the likelihood of death at hospital discharge (weighted risk ratio = 1.91, 95% CI 0.91 − 3.68). In subgroup analysis, the possibility of good functional recovery was lower in patients with intracerebral hemorrhage (weighted risk ratio = 0.88, 95% CI 0.79 − 0.98; *P* for interaction = 0.01) and seizures (weighted risk ratio = 0.86, 95% CI 0.76 − 0.95; *P* for interaction = 0.03).

**Conclusion:**

In this large nationwide study, EVT was not associated with improved functional outcomes compared to standard care in patients with CVT.

**Supplementary Information:**

The online version contains supplementary material available at 10.1186/s40779-025-00605-3.

## Background

Cerebral venous thrombosis (CVT) is an uncommon type of stroke [1, 2], with higher incidence in women and usually occurring during pregnancy [3]. Anticoagulation is the mainstay of treatment, even if intracerebral hemorrhage (ICH) is present [4]. Although outcomes from CVT are better than for numerous other neurovascular emergencies, death still occurs in up to 10% of cases and many patients fail to make a complete recovery [5]. Endovascular treatment (EVT) is therefore gaining popularity for managing severe cases of CVT, although the evidence for safety and efficacy is limited [6, 7]. The only randomized controlled trial, the thrombolysis or anticoagulation for CVT, was prematurely terminated due to ineffectiveness after only 67 patients were recruited [8]. Herein, we present the results of a large observational study that aimed to compare the outcomes of patients with CVT who received EVT in addition to optimal medical care versus those who received optimal medical care alone.

## Methods

### Overview

The multicenter registry study of CVT in China was established to determine the profile, management and outcome of hospitalized patients with CVT from January 2018 to June 2022. Hospitals were chosen to be broadly representative of the range of patients affected by CVT in China. The study included provincial and prefecture-level hospitals according to patient volumes and clinical capacity, as CVT is rare and often misdiagnosed in small county-level hospitals. This study was registered at ClinicalTrials.gov (NCT05448248).

In short, our randomization method treats each province as a unit. The first step involves selecting cities: each province chooses 1 capital city and 3 prefecture-level cities. To select the 3 prefecture-level cities, we rank all prefecture-level cities within the province based on their 2021 gross domestic product per capita levels, then divide them into 3 categories: high, medium, and low. One city is randomly selected from each of these 3 categories. Accordingly, each province drew 4 cities, including 1 provincial capital and 3 prefecture-level. The second step is to select hospitals: for each chosen city, we compile a list of all tertiary hospitals and randomly select 1 hospital from each city. This method ensures that the selected hospitals are representative of the province, considering both geographic and economic factors. A total of 104 hospitals were selected to participate in the study. This study was designed in accordance with the STROBE criteria [9]. The protocol has been published elsewhere [10]. The study was centrally approved by the ethics committee of Hunan Brain Hospital (Z2017006).

### Patient enrollment

Patients in each hospital were identified if they met the following criteria: diagnosis of CVT according to the International Classification of Diseases (ICD), initially using ICD-9 codes (325.0, 437.6, and 671.5) and then ICD-10 codes (I63.6, I67.6, O22.5X, O87.3, or G08) [11], which was confirmed upon review of medical records and imaging studies. In this study, “standard care” was defined as treatment in which patients did not receive EVT but were treated with conventional anticoagulation therapy and other standard clinical interventions. Each patient had a different interval from discharge to the current follow-up, therefore, the follow-up duration was variable and depended on the discharge date leading up to the present. Informed consent was obtained from each patient or legally authorized representative (primarily a spouse, parent, adult child, or otherwise as indicated).

### Data collection

**Fig. 1:**
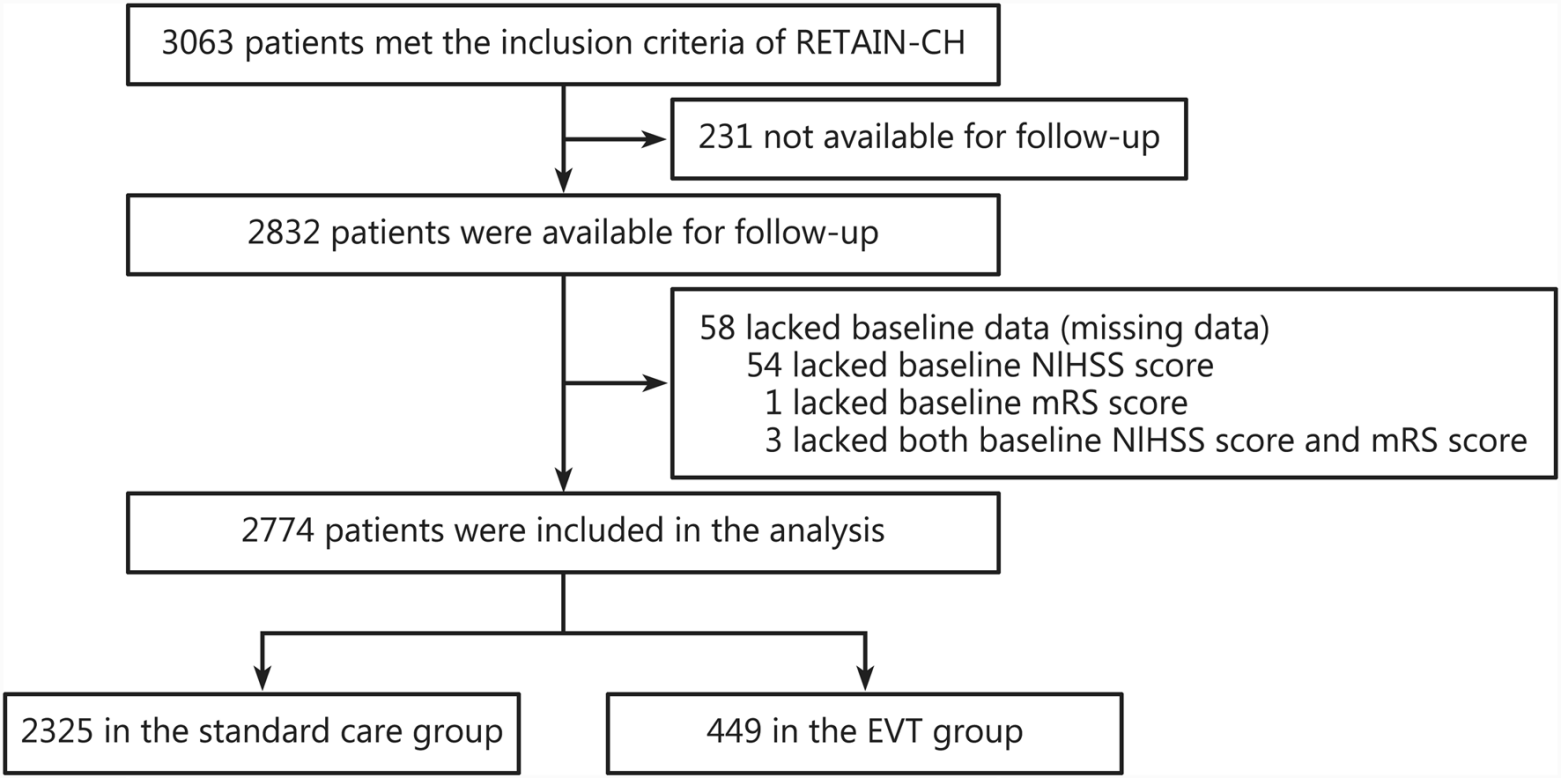
Study flow chart. RETAIN-CH multicenter registry study of cerebral venous thrombosis in China, NIHSS National Institutes of Health Stroke Scale, mRS modified Rankin Scale, EVT endovascular treatment

All data were collected electronically in a standardized format using a secure bespoke database on the Bigdata Observatory platform for stroke in China [12], with multiple levels of quality control. Research staff at the participating hospital were trained in the use of a standardized protocol and formally certified before the study commencement. The training included methods and criteria to identify cases of CVT from their respective hospital database, and completion of data entry and follow-up assessments. Each participating hospital had a professional data manager to verify and monitor the quality and integrity of the data. Research staff contacted eligible patients to ascertain their vital status and level of recovery either through face-to-face or telephone interviews. Regarding missing data, the study had 58 patients with missing baseline National Institutes of Health Stroke Scale (NIHSS) scores or modified Rankin Scale (mRS) scores (Fig. 1). Due to the limited number of missing values, these patients were excluded from the analysis.

### Outcomes

The primary outcome was the proportion of patients recovering without disability (mRS 0 − 1). Secondary outcomes included the proportion of patients with mRS scores 0 − 2 or 0 − 3 at the time of hospital discharge and follow-up. Safety outcomes included all-cause mortality at hospital discharge and follow-up.

### Statistical analysis

The characteristics of patients who received EVT were expected to differ from those of the standard care group. To generate a comparable dataset, we calculated a propensity score to estimate the individual probability of a patient receiving endovascular therapy. Eighteen variables were used as covariates to calculate propensity scores: sex, age (continuous), vomiting, seizure, reduced level of consciousness, coma, motor deficit, sensory deficit, hypertension, cerebral hemorrhage, subarachnoid hemorrhage, pulmonary infection, warfarin, rivaroxaban, baseline neurological severity according to scores on the NIHSS (0 − 1, 2 − 4, ≥ 5) and the mRS (0 − 1 versus 2 − 5), hospital level, follow-up time (time from symptom onset to last contact; continuous). An inverse probability of treatment weighting (IPTW) was utilized as the primary strategy to adjust for imbalances in the baseline characteristics [13]. Data balancing was examined using an absolute standardized difference in covariate means. Stabilized weights were employed to reduce the variance in the estimated effect of EVT, and incorporated into modified Poisson regression models to determine associations of EVT and outcome [13]. The effects were expressed as a weighted risk ratio and 95% confidence interval (CI).

We conducted a sensitivity analysis around the propensity score matching, details of which are provided in the Additional file 1: Methods. All *P* values are two-sided, with values < 0.05 considered to indicate statistical significance. All statistical analyses were performed by using R software, version 4.3.2.

## Results

### Characteristics of participants

Of 3063 patients identified through screening, 2774 were available for follow-up and included in these analyses, of whom 449 (16.2%) were treated with EVT and 2325 (83.8%) with standard care (Fig. 1; Additional file 1: Fig. S1). Their mean age was 42 [standard deviation (SD) 15.8] years, 50.3% were female, and the baseline median NIHSS was 0 [interquartile range (IQR) 0 − 1]. A total of 231 patients were lost to follow-up, primarily due to incorrect contact information or the inability to reach the patients after multiple attempts (Fig. 1). Table 1 outlines the baseline characteristics of the participants according to treatment. Compared to those receiving standard care, participants who underwent EVT were younger and more likely to exhibit neurological symptoms related to raised intracranial pressure, such as vomiting, seizures, and loss of consciousness, and to have experienced motor or sensory neurological impairment. Additionally, those who received EVT were more likely to have a complication of CVT, such as ICH, subarachnoid hemorrhage, or pulmonary infection. Participants who underwent EVT had greater neurological impairment (NIHSS scores ≥ 5) or disability (scores 2 − 5 on the mRS) at presentation than the other participants. The distribution of baseline covariates was fairly well balanced between the groups after the application of propensity scores, with the absolute standardized differences after IPTW within an acceptable margin of 0.1 (Table 1; Additional file 1: Fig. S2).

**Table 1:** Baseline characteristics of patients with cerebral venous thrombosis (CVT) by use of endovascular treatment (EVT)^a^

Characteristic	All patients			Propensity score weighted patients^b^		
	EVT (*n* = 449)	Standard care (*n* = 2325)	*P*-value	EVT (*n* = 441.7)	Standard care (*n* = 2327.9)	SMD^c^
Age (years, mean ± SD)	38.7 ± 13.7	43.0 ± 15.5	< 0.01	41.6 ± 14.0	42.3 ± 15.4	0.04
Female [*n* (%)]	224 (49.9)	1172 (50.4)	0.88	233.5 (52.9)	1176.9 (50.6)	0.04
Symptoms [*n* (%)]						
Vomiting	200 (44.5)	857 (36.9)	< 0.01	179.5 (40.6)	890.3 (38.2)	0.05
Seizure	108 (24.1)	393 (16.9)	< 0.01	75.7 (17.1)	418.0 (18.0)	0.02
Loss of consciousness	120 (26.7)	353 (15.2)	< 0.01	77.2 (17.5)	396.8 (17.0)	0.02
Motor deficit	141 (31.4)	444 (19.1)	< 0.01	94.8 (21.5)	492.4 (21.2)	0.01
Sensory deficit	62 (13.8)	145 (6.2)	< 0.01	37.0 (8.4)	174.3 (7.5)	0.04
History of hypertension [*n* (%)]	66 (14.7)	442 (19.0)	0.04	79.7 (18.1)	425.0 (18.3)	0.01
Complications [*n* (%)]						
Intracerebral hemorrhage	129 (28.7)	408 (17.5)	< 0.01	83.6 (18.9)	451.5 (19.4)	0.01
Subarachnoid hemorrhage	58 (12.9)	159 (6.8)	< 0.01	38.1 (8.6)	185.6 (8.0)	0.02
Pulmonary infection	61 (13.6)	171 (7.4)	< 0.01	41.0 (9.3)	196.9 (8.5)	0.03
Drug [*n* (%)]						
Warfarin	215 (47.9)	780 (33.6)	< 0.01	154.9 (35.1)	834.9 (35.9)	0.03
Rivaroxaban	43 (9.6)	374 (16.1)	< 0.01	75.5 (17.1)	350.1 (15.0)	0.06
Baseline NIHSS score [*n* (%)]			< 0.01			0.02
0 − 1	295 (65.7)	1806 (77.7)		332.8 (75.4)	1761.3 (75.7)	
2 − 4	72 (16.0)	301 (12.9)		61.6 (13.9)	315.7 (13.6)	
≥ 5	82 (18.3)	218 (9.4)		47.3 (10.7)	251.0 (10.8)	
Baseline mRS score^d^ [*n* (%)]			< 0.01			0.05
0 − 1	263 (58.6)	1597 (68.7)		287.0 (65.0)	1559.3 (67.0)	
2 − 5	186 (41.4)	728 (31.3)		154.7 (35.0)	768.6 (33.0)	
Hospital level [*n* (%)]			< 0.01			0.02
Provincial hospital	305 (67.9)	1102 (47.4)		230.5 (52.2)	1184.5 (50.9)	
Prefecture-level hospital	144 (32.1)	1223 (52.6)		211.1 (47.8)	1143.4 (49.1)	
Mean follow-up^e^ [days, mean ± SD]	822.3 ± 492.4	902.4 ± 507.3	< 0.01	887.3 ± 508.2	889.8 ± 507.1	0.01

^a^EVT includes use of intra-arterial thrombolysis, intrasinus thrombolysis, intrasinus stenting, and mechanical thrombectomy. ^b^Propensity score were weighted using inverse probability of exposure weighting. ^c^The difference between groups divided by SMD, a value > 10% is interpreted as a meaningful difference. ^d^Scores on the mRS for functional recovery range from 0 to 6, with higher scores indicating more severe disability and 6 indicating death. ^e^Follow-up refers to the time from hospital discharge to the date of assessment

*SMD* standardized mean difference,* NIHSS* National Institutes of Health Stroke Scale,* mRS* modified Rankin Scale

Participants who received EVT in provincial hospitals were younger than those in prefecture-level hospitals, but they had less severe neurological and functional severity (Additional file 1: Table S1). Additional file 1: Table S2 highlights the diverse EVT methods employed in this study. The most commonly used method was mechanical thrombectomy (MT), performed in 30.10% of patients who underwent EVT, followed by intrasinus thrombolysis (IT, 29.60%) and a combination of MT and IT (19.80%). In prefecture-level hospitals, participants were more likely to receive MT alone (42.40%) and had less IT (22.20%) (Additional file 1: Table S2).

### Primary and secondary outcomes

The distribution of scores on the mRS at present is shown in Additional file 1: Fig. S3. Compared to the standard care group, those who received EVT had a significantly lower likelihood of good functional recovery (mRS 0 − 1) at present (risk ratio = 0.93, 95% CI 0.90 − 0.97) (Table 2). However, the strength of this association became non-significant after multivariable adjustment (risk ratio = 0.98, 95% CI 0.95 − 1.02) and with the use of IPTW (risk ratio = 1.00, 95% CI 0.96 − 1.03) (Table 2). These results were consistent in sensitivity analysis with propensity score-matching with a risk ratio of 0.97 (95% CI 0.92 − 1.03). Neutral associations who also found between EVT and secondary outcomes (Additional file 1: Tables S3, S4). The most commonly used EVT method was MT (Additional file 1: Table S2). A subgroup analysis was conducted based on EVT methods (MT versus other methods), and the results showed no significant difference (Additional file 1: Table S5).

**Table 2:** Endovascular treatment (EVT) and outcomes among patients with cerebral venous thrombosis (CVT)^a^

Outcome	EVT [*n* = 449, *n* (%)]	Standard care [*n* = 2325, *n* (%)]	Unweighted patients		Propensity score weighted patients
			Unadjusted risk ratio (95% CI)	Adjusted risk ratio (95% CI)^b^	Weighted risk ratio (95% CI)^c^
Primary outcome					
mRS score at present^d^					
0 − 1	379 (84.4)	2102 (90.4)	0.93 (0.90 − 0.97)	0.98 (0.95 − 1.02)	1.00 (0.96 − 1.03)
2 − 6	70 (15.6)	223 (9.6)	1.00	1.00	1.00
Secondary outcomes					
mRS score at present					
0 − 2	404 (90.0)	2178 (93.7)	0.96 (0.93 − 0.99)	1.00 (0.97 − 1.03)	1.01 (0.98 − 1.03)
3 − 6	45 (10.0)	147 (6.3)	1.00	1.00	1.00
mRS score at present					
0 − 3	414 (92.2)	2203 (94.8)	0.97 (0.95 − 1.00)	1.01 (0.98 − 1.03)	1.01 (0.98 − 1.03)
4 − 6	35 (7.8)	122 (5.2)	1.00	1.00	1.00
mRS score at discharge					
0 − 1	300 (66.8)	1848 (79.5)	0.84 (0.79 − 0.89)	0.94 (0.89 − 1.00)	0.94 (0.89 − 0.99)
2 − 6	149 (33.2)	477 (20.5)	1.00	1.00	1.00
mRS score at discharge					
0 − 2	349 (77.7)	2063 (88.9)	0.88 (0.84 − 0.91)	0.96 (0.92 − 1.00)	0.95 (0.91 − 0.99)
3 − 6	100 (22.3)	262 (11.1)	1.00	1.00	1.00
mRS score at discharge					
0 − 3	380 (84.6)	2148 (92.4)	0.92 (0.89 − 0.95)	0.99 (0.96 − 1.03)	0.99 (0.96 − 1.02)
4 − 6	69 (15.6)	177 (7.6)	1.00	1.00	1.00
Safety outcomes					
Death at discharge	18 (4.0)	24 (1.0)	3.88 (2.09 − 7.09)	1.59 (0.64 − 3.25)	1.91 (0.91 − 3.68)
Survival at discharge	431 (96.0)	2301 (99.0)	1.00	1.00	1.00
Safety outcomes					
Death at present	23 (5.1)	77 (3.3)	1.55 (0.96 − 2.40)	1.02 (0.63 − 1.61)	0.92 (0.52 − 1.54)
Survival at present	426 (94.9)	228 (96.7)	1.00	1.00	1.00

^a^EVT includes use of intra-arterial thrombolysis, intrasinus thrombolysis, intrasinus stenting, mechanical thrombectomy. ^b^Adjustments were made for age, sex (male or female), symptoms (vomiting, seizure, consciousness disturbance, motor deficit, sensory deficit, hypertension), diagnosis (cerebral hemorrhage, subarachnoid hemorrhage, pulmonary infection), drug therapy in hospital (warfarin, rivaroxaban), baseline NIHSS score (0 − 1, 2 − 4 or ≥ 5), baseline mRS score (0 − 1or 2 − 5), and follow-up time. ^c^Shown is the risk ratio from a modified Poisson regression model and covariates with inverse probability weighting according to the propensity score. ^d^Scores on the mRS of functional recovery range from 0 to 6, with higher scores indicating more severe disability and 6 indicating death

*CI* confidence interval,* NIHSS* National Institutes of Health Stroke Scale,* mRS* modified Rankin Scale

### Safety outcomes

Mortality at discharge was 4.0% in the EVT group and 1.0% in the standard care group. Compared with standard care, EVT was associated with an increased likelihood of death at discharge (unadjusted risk ratio = 3.88, 95% CI 2.09 − 7.09) in unadjusted analysis, but was non-significant after multivariable adjustment (adjusted risk ratio = 1.59, 95% CI 0.64 − 3.25) and IPTW (weighted risk ratio = 1.91, 95% CI 0.91 − 3.68) (Table 2). Mortality occurred in 5.1% in the EVT group and 3.3% in the standard care group at present (Table 2).

**Fig. 2:**
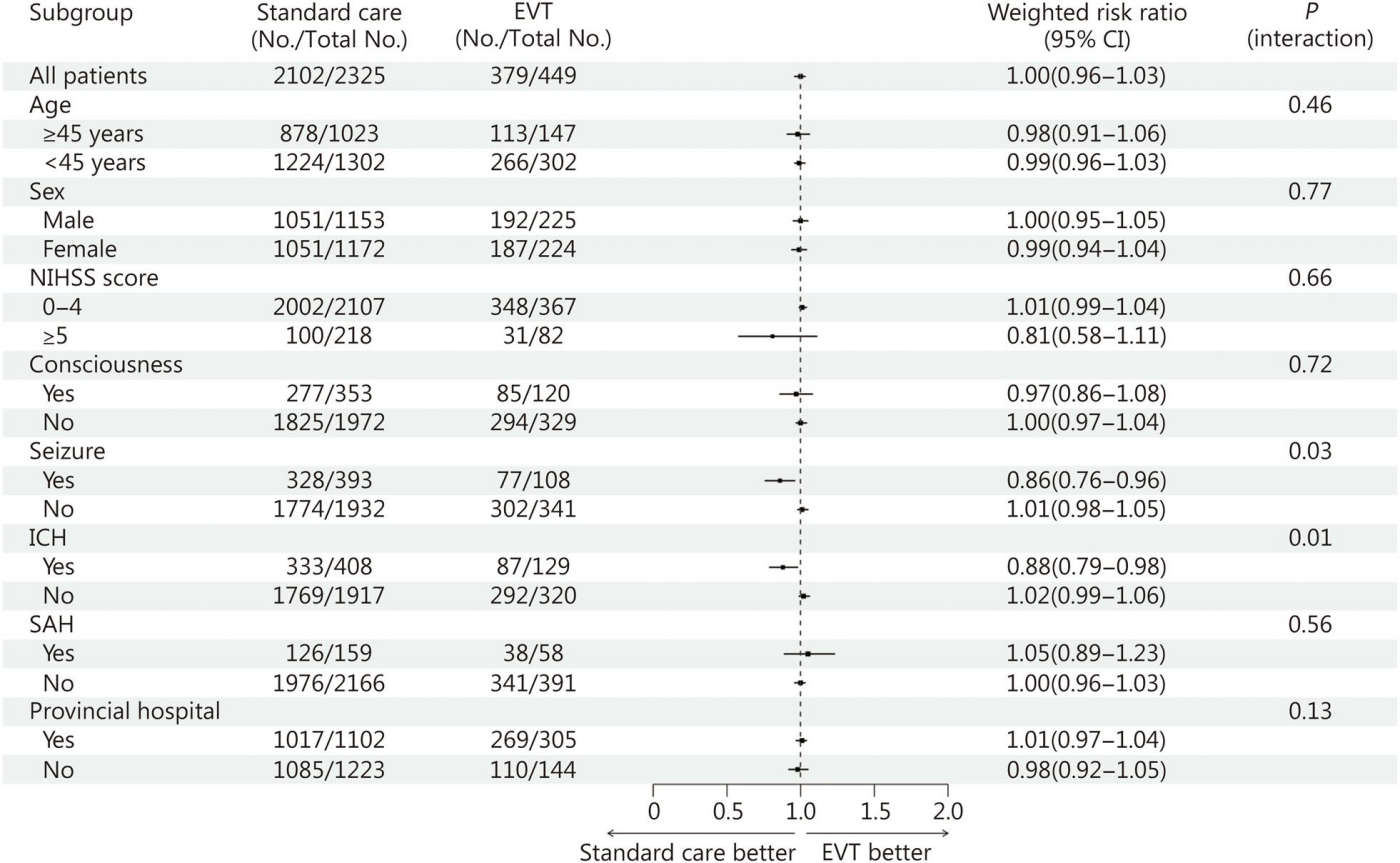
Subgroup analyses of endovascular treatment (EVT) for cerebral venous thrombosis (CVT). Scores on the modified Rankin Scale (mRS) of functional recovery range from 0 to 6, with higher scores indicating more severe disability and 6 indicating death. Scores on National Institutes of Health Stroke Scale (NIHSS) range from 0 to 42, with higher scores indicating greater neurologic deficits. CI confidence interval, ICH intracranial hemorrhage, SAH subarachnoid hemorrhage

Figure 2 shows that the associations of EVT with the outcomes were consistent across the key subgroups by age, sex, and stroke severity. However, significant heterogeneity was apparent for patients with CVT who had early ICH, where endovascular therapy was associated with poor functional recovery (weighted risk ratio = 0.88, 95% CI 0.79 − 0.98; *P* for interaction = 0.01) (Fig. 2). EVT was also associated with a poor functional recovery in those patients who presented with a seizure (weighted risk ratio = 0.86, 95% CI 0.76 − 0.96; *P* for interaction = 0.03) (Fig. 2).

## Discussion

In this nationwide representative study of CVT, we have shown that EVT was not associated with improved functional recovery compared to standard care. There was also no significant difference in mortality between patients receiving EVT and standard care, suggesting the intervention is safe when involving MT with or without IT in these patients.

Our main findings align with those of the only randomized controlled trial on this topic, where EVT was found to be feasible and safe in a highly selected patient cohort but without an effect on functional outcome. However, compared to participants in thrombolysis or anticoagulation for CVT [8] who had a median NIHSS score of 12 (IQR 7 − 20), our registry included patients with less severe neurological impairment, where 65.7% of those who received EVT having an NIHSS score of 0 − 1 (Table 1**)**. The findings are more relevant to the broader profile of patients with CVT who are less neurologically severe. While some observational studies have demonstrated the safety and feasibility of EVT in selected patients and shown promising results, the lack of a control group, retrospective nature, or insufficient statistical power has limited the ability to demonstrate a clear treatment effect for benefit [14–16]. This large national representative sample addressed many of these limitations and the use of propensity score analysis reduced the influence of confounding imbalance. The intent to randomly select 104 participating hospitals across 31 provinces and municipalities in China, and taking into account variable socioeconomic status, population size, and hospital type, was to enhance the representativeness of study population and generalizability of findings. The prospective collection of outcomes in a systematic manner minimized the potential for recall bias.

The safety of EVT for patients with CVT has been a controversial issue. Previous large-scale epidemiological studies and meta-analyses have suggested that EVT is associated with an increased risk of death [17]. However, this could be attributed to indication bias where EVT is undertaken in more severe cases who do not respond to, or worsen despite, anticoagulant therapy [18]. Moreover, patients with worse clinical conditions upon admission are more likely to receive aggressive treatment and have poorer outcomes, regardless of the treatment administered. In our large representative study, EVT was not associated with an increase in mortality, both during hospitalization and after discharge, suggesting that EVT is a safe strategy for patients with CVT.

In subgroup analysis, we observed a relatively poorer therapeutic effect of EVT for patients with seizures or ICH. This finding is crucial as it provides a theoretical basis for selecting patients who may benefit from EVT. In clinical practice, patients with ICH or epilepsy associated with CVT are considered to be severe cases, and to have a poor prognosis. ICH has been identified as an independent risk factor for poor outcomes in endovascular-treated patients [5, 19]. Additionally, EVT is invasive and increases the risk of hemorrhage, which may complicate recovery. In our study, the primary focus was on ICH before EVT. Epilepsy is also a manifestation of severe CVT, potentially related to cerebral infarction/edema, or cortical vein thrombosis [20].

In our study population, the predominant EVT modalities were MT, IT, or their combination, which is reflective of clinical practice in China. We also observed variability between different levels of hospitals. Compared with provincial hospitals, patients in prefecture-level hospitals were more likely to receive MT and less likely to undergo IT and intrasinus stenting. These differences may indicate variations in EVT technology and treatment philosophy between provincial and municipal hospitals.

This study had some limitations. First, the patient characteristics, treatments, and outcomes at discharge included were obtained from medical records retrospectively, and there may be undocumented information. Second, the timing of receiving EVT may influence the outcomes. However, this was not available in the medical records and was not taken into consideration in this study. Third, details on the EVT procedure (used devices, radiological outcome, complications) were not reported.

## Conclusions

In this large nationwide study, we found that EVT did not improve functional recovery compared to standard care. However, EVT was safe for patients with CVT without increasing the risk of mortality. For CVT patients presenting with ICH and seizure, EVT was associated with a worse functional recovery. This study underscores the need for cautious EVT application in CVT in the absence of robust supporting evidence.

## Supplementary Information

**Additional file 1.** List of multicenter registry study of cerebral venous thrombosis in China (RETAIN-CH) investigators and participation center. Methods. **Table S1** Characteristics of patients by different levels of hospitals. **Table S2** Components of endovascular treatment (EVT) for the patients (%). **Table S3** Characteristics of patients, before and after propensity score matching. **Table S4** Sensitivity analyses of primary, secondary outcomes, and safety outcomes using propensity score matching. **Table S5** Different endovascular treatment (EVT) and outcomes among patients with cerebral venous thrombosis (CVT). **Fig. S1** Study flow chart timeline. **Fig. S2** Standardized mean differences in the unweighted and weighted patients. **Fig. S3** Distribution of the modified Rankin Scale (mRS) score at present in all patients.

## Data Availability

The data utilized for this analysis can be obtained by contacting the corresponding author via email. We encourage interested parties to reach out for access to the dataset.

## Abbreviations

CI: Confidence intervals
CVT: Cerebral venous thrombosis
EVT: Endovascular treatment
ICD: International classification of diseases
ICH: Intracerebral hemorrhage
IT: Intrasinus thrombolysis
IPTW: Inverse probability of treatment weighting
mRS: Modified Rankin Scale
MT: Mechanical thrombectomy
NIHSS: National Institutes of Health Stroke Scale
