# Supplementary material for: Endovascular treatment for cerebral venous thrombosis: a multicenter study in China
Source: Mil Med Res. 2025 Apr 8;12:16. doi: 10.1186/s40779-025-00605-3 (PMC11978133; doi:10.1186/s40779-025-00605-3)
Supplement: Supplementary file 1 — Additional file 1. List of multicenter registry study of cerebral venous thrombosis in China (RETAIN-CH) investigators and participation center. Methods. Table S1 Characteristics of patients by different levels of hospitals. Table S2 Components of endovascular treatment (EVT) for the patients (%). Table S3 Characteristics of patients, before and after propensity score matching. Table S4 Sensitivity analyses of primary, secondary outcomes, and safety outcomes using propensity score matching. Table S5 Different endovascular treatment (EVT) and outcomes among patients with cerebral venous thrombosis (CVT). Fig. S1 Study flow chart timeline. Fig. S2 Standardized mean differences in the unweighted and weighted patients. Fig. S3 Distribution of the modified Rankin Scale (mRS) score at present in all patients. [file 40779_2025_605_MOESM1_ESM.pdf]

## List of multicenter registry study of cerebral venous thrombosis in China (RETAIN-CH) investigators and participation center

**The investigators' full names and academic degrees are as follows:**

Xun-Ming Ji, M.D., Ph.D., Craig S. Anderson, M.D., Ph.D., Xia Wang, Ph.D., He-Tao Bian, M.D., Gui-You Liu, Ph.D., Chen Zhou, M.D., Ran Meng, M.D., Lan Liu, Ph.D., Jian-Gang Duan, M.D., Feng Yan, M.D., Chuan-Hui Li, M.D., Min Li, M.D., Wen Hui, Ph.D., Xu-Xiang Zhang, M.D., Ph.D., Dong Zhao, M.D., Ph.D., Ya-Peng Li, M.D., Qi Fang, M.D., De-Zhi Kang, M.D., Hong-Liang Zeng, M.D., Zhi-Jian Liang, M.D., Zheng-Hao Shi, M.D., Wei Yue, M.D., Qin-Jian Sun, M.D., Gui-Sheng Chen, M.D., Jian-Long Song, M.D., Zhong-Rui Yan, M.D., Qiu-Hong Ji, M.D., Kai-Jie Wang, M.D., Lu-Sha Tong, M.D., Xiao Hu, M.D., Wen-Feng Cao, M.D., Wei Yan, M.D., Rui-Jiang Gao, M.D., Qi Li, M.D., Jian-Yi Wang, M.D., Yi Liu, M.D., Bao-Jun Wang, M.D., Xiao-Hua Wang, M.D., Sheng-Tao Yao, M.D., Ye Lang, M.D., Hai-Peng Li, M.D., Xing-Yu Chen, M.D., Hong Liu, M.D., Jing-Wei Li, M.D., You-Quan Gu, M.D., Yan-Bo Cheng, M.D., Wen-Li Li, M.D., Xiang-Jian Xiang, M.D., Chun-Ling Li, M.D., Jia Ke, M.D., Li-Hua Wang, M.D., Jing-Jun Feng, M.D., Ya-Nan Zheng, M.D., Feng Tian, M.D., Ying Zeng, M.D., Yan-Qin Zeng, M.D., Shi-Jin Ning, M.D., Yue-Ling Li, M.D., You-Ting Zhou, M.D., Fu-Qiang Guo, M.D., Xiao-Rong Dai, M.D., Shi-Bin Wen, M.D., Shao-Fa Li, M.D., Yong Deng, M.D., Zheng-Hao Shi, M.D., Ying Chen, M.D., Wei Xu, M.D., Wen-Jie Xiang, M.D., Pei-Yang Zhou, M.D., Qiang Liu, M.D., Xue-Gang Meng, M.D., Peng-Cheng Fu, M.D., Huan-Dong Liu, M.D., Guo-Ping Ma, M.D., Xin-Hua Liu, M.D., Qing-Hua Liu, M.D., Xue-Ying Shi, M.D., Xi-Peng Li, M.D., Xiao-Ling Guo, M.D., Jie Zhang, M.D., Xi-Ai Yang, M.D., Jia-Liang Zhang, M.D., Xiao-Xiang Peng, M.D., Zhi-Ying Chen, M.D., Bin Chen, M.D., Yu-Jie Jia, M.D., Yu-Ying Li, M.D., Yan Ma, M.D., Mei Yang, M.D., Zhao-Hai Feng, M.D., Jing-Lei Zhu, M.D., Jiang-Tao Zhang, M.D., Cui-Hua Li, M.D., Xin-Pu Zhu, M.D., Xiang Fang, M.D., Tie-Yan Wang, M.D., Xiu-Fen Zhang, M.D., Xuan Cui, M.D., Cheng-He Sun, M.D., Da-Chao Li, M.D., Ji-Long Cheng, M.D., Xiao-Hua Yu, M.D., Long Ma, M.D., Qi Han, M.D., Yi-Fei Ji, M.D., Bing Xu, M.D., Ying-Jing Yang, M.D., Zhi-Bin Zhou, M.D., Bing Zhang, M.D., Wen-Biao Guan, M.D., Ting-Yi Li, M.D., Lina Wang, M.D., Li-Jing Zhang, M.D., Hu Wang, M.D., Tao Qiu, M.D., Chun-Lei He, M.D.

**The investigators' affiliations are as follows:**

(XMJ) Xuanwu Hospital Capital Medical University, Beijing, China; (CSA) the George Institute for Global Health, University of New South Wales, Sydney, NSW, Australia; (HTB) Xuanwu Hospital

Capital Medical University, Beijing, China; (XW) the George Institute for Global Health, University of New South Wales, Sydney, NSW, Australia; (YPL) the First Affiliated Hospital of Zhengzhou University, Shanghai, China; (QF) the First Affiliated Hospital of Soochow University, Suzhou, China; (DZK) the First Affiliated Hospital of Fujian Medical University, Fujian, China; (HLZ) Ganzhou People's Hospital, Ganzhou, China; (ZJL) the First Affiliated Hospital of Guangxi Medical University, Nanning, China; (ZHS) the First Affiliated Hospital of Wenzhou Medical University, Wenzhou, China; (WY) Tianjin Huanhu Hospital, Tianjin, China; (QJS) Shandong Provincial Hospital Affiliated to Shandong First Medical University, Jinan, China; (GSC) General Hospital of Ningxia Medical University, Yinchuan, China; (JLS) the First Affiliated Hospital of USTC, Hefei, China; (ZRY) Jining First People's Hospital, Jining, China; (QHJ) Affiliated Hospital of Nantong University, Nantong, China; (KJW) Tangshan Gongren Hospital Tangshan, China; (LST) the Second Affiliated Hospital of Zhejiang University School of Medicine, Hangzhou, China; (XH) Guizhou Provincial People's Hospital, Guiyang, China; (WFC) Jiangxi Provincial People's Hospital, Nanchang, China; (WY) the First People's Hospital of Kashi; (RJG) Inner Mongolia People's Hospital, Hohhot, China; (QL) The Second Affiliated Hospital of Anhui Medical University, Hefei, China; (JYW) the First Affiliated Hospital of Xi'an Jiaotong University, Xian, China; (YL) Shanxi Provincial People's Hospital, Taiyuan, China; (BJW) Baotou Central Hospital Inner Mongolia, Baotou, China; (XHW) Qujing NO.1 hospital, Qujing, China; (STY) Affiliated Hospital of Zunyi Medical University, Zunyi, China; (YL) Shengli oilfield central hospital, Dongying, China; (HPL) the First People's Hospital of Chenzhou, Chenzhou, China; (XYC) Zhongshan Hospital of Xiamen University, Xiamen, China; (HL) Heping Hospital Affiliated to Changzhi Medical College, Changzhi, China; (JWL) Nanjing Drum Tower Hospital, Nanjing, China; (YQG) the First School of Clinical Medicine, Lanzhou University, Lanzhou, China; (YBC) Affiliated Hospital of Xuzhou Medical University, Xuzhou, China; (WLL) Chifeng Municipal Hospital, Chifeng, China; (XJX) Hebei general hospital, Shijiazhuang, China; (CLL) Central People's Hospital of Zhanjiang, Zhanjiang, China; (JK) Zhumadian central hospital, Zhumadian, China; (LHW) the Second Affiliated Hospital of Harbin Medical University, Harbin, China; (JJF) Liaocheng People's Hospital, Liaocheng, China; (YAZ) Ordos Central Hospital, Ordos, China; (FT.) the First Affiliated Hospital of Shihezi University, Shihezi, China; (YZ) Yichun People's Hospital, Yichun, China; (YQZ) Longyan First Hospital, Lonyan, China; (SJN) the Second People's Hospital Medical Group of Qinzhou, Qinzhou, China; (YLL) Luohe Central Hospital, Luohe, China; (YTZ) Qinghai Provincial People's Hospital, Xining, China; (FQG) Sichuan Provincial People's Hospital, Chengdu, China; (XRD) the People's Hospital of Yuxi City, Yuxi, China; (SBW) Jiuquan City People's Hospital, Jiuquan, China; (SFL) the People's Hospital of Baise, Baise, China; (YD) Dalian Municipal Central Hospital, Dalian, China; (YC) Xingyi People's Hospital, Xingyi,

China; (WX) the Affiliated Changsha Central Hospital, Changsha, China; (WJX) 3201 Hospital of Xi'an Jiaotong University Health Science Center, Hanzhong, China; (PYZ) Xiangyang No. 1 People's Hospital, Xiangyang, China; (QL) Yan'an university affiliated hospital, Yanan, China; (XGM) People's Hospital of Xinjiang Uygur Autonomous Region, Urumchi, China; (PCF) Shenzhen Second People's Hospital, Shenzhen, China; (HDL) the People's Hospital of Xizang Autonomous Region, Lhasa, China; Shaoxing People's Hospital, Shaoxing, China; (GPM) Tian Shui First People's Hospital, Tianshui, China; (XHL) the Third Affiliated Hospital of Guangzhou Medical University, Guangzhou, China; (QHL) Meizhou People's Hospital, Meizhou, China; (XYS) Anqing Municipal Hospital, Anqing, China; (XPL) Xingtai People's Hospital, Xingtai, China; (XLG) First Affiliated Hospital of Anhui University of Science and Technology, Huainan, China; (JZ) the Second Affiliated Hospital of Kunming Medical University, Kunming, China; (XAY) Ankang Central Hospital, Ankang, China; (JLZ) Xinyang Central Hospital, Xingyang, China; (XXP) Hubei Third People's Hospital, Wuhan, China; (ZYC) Affiliated Hospital of Jiujiang University, Jiujiang, China; (BC) the Second Affiliated Hospital of Hainan Medical University, Haikou, China; (YJJ) the First Affiliated Hospital of Jinzhou Medical University, Jinzhou, China; (YYL) Wuzhou Workers' Hospital, Wuzhou, China; (YM) Yueyang Central Hospital, Yueyang, China; (MY) Dali Nationality Autonomous Prefecture Hospital, Dali, China; (ZHF) Maanshan People's Hospital, Maanshan, China; (JLZ) Dandong Central Hospital, Dandong, China; (JTZ) Chengde Central Hospital, Chengde, China; (CHL) Linfen Central Hospital, Linfen, China; Jinzhong First People's Hospital, Jinzhou, China; (XPZ) Tongji University Affiliated Shanghai Fourth People's Hospital, Shanghai, China; (XF) the First Hospital of Nanping City, Nanping, China; (TYW) Daqing Oilfield General Hospital, Daqing, China; (XFZ) Jilin Province People's Hospital, Changchun, China; (XC) the Second People's Hospital Of Mudanjiang City, Mudanjiang, China; (CHS) the First Hospital of Qiqihar, Qiqihar, China; (DCL) Tonghua Central Hospital, Tonghua, China; (JLC) Karamay Central Hospital, Karamay, China; (XHY) Shannan People's Hospital of Tibet, Shannan, China; (LM) Xining No.1 People's Hospital, Xining, China; (QH) Sinopharm Dongfeng General Hospital, Shiyan, China; (YFJ) the Second Clinical College of North Sichuan Medical College, Nanchong, China; (BX) Shenyang Tenth People's Hospital, Shenyang, China; Hunan Brain Hospital, Changsha, China; (YJY) the Second Affiliated Hospital of Guizhou Medical University, Kaili, China; (ZBZ) Sanya People's Hospital, Sanya, China; (BZ) Huzhou Central Hospital, Huzhou, China; (WBG) Baiyin First People's Hospital, Baiyin, China; (TYL) Xiaogan Central Hospital, Xiaogan, China; (LNW) Jilin City Central Hospital, Jilin, China; (LJZ) Siping City Central Hospital, Siping, China; (HW) Wuzhong City People's Hospital, Wuzhou, China; (TQ) Zigong First People's Hospital, Zigong, China; (CLH) Suining Central Hospital, Suining, China.

**The names of the participating centers are as follows:**

Xuanwu Hospital, Capital Medical University; the First Affiliated Hospital of Zhengzhou University; the First Affiliated Hospital of Soochow University; the First Affiliated Hospital of Fujian Medical University; Ganzhou People's Hospital; the First Affiliated Hospital of Guangxi Medical University; the First Affiliated Hospital of Wenzhou Medical University; Tianjin Huanhu Hospital; Shandong Provincial Hospital Affiliated to Shandong First Medical University; General Hospital of Ningxia Medical University; the First Affiliated Hospital of USTC; Jining First People's Hospital; Affiliated Hospital of Nantong University; Tangshan Gongren Hospital; the Second Affiliated Hospital of Zhejiang University School of Medicine; Guizhou Provincial People's Hospital; Jiangxi Provincial People's Hospital; the First People's Hospital of Kashi; Inner Mongolia People's Hospital; the Second Affiliated Hospital of Anhui Medical University; the First Affiliated Hospital of Xi'an Jiaotong University; Shanxi provincial People's hospital; Baotou Central Hospital Inner Mongolia; Qujing No. 1 Hospital; Affiliated Hospital of Zunyi Medical University; Shengli Oilfield Central Hospital; the First People's Hospital of Chenzhou; Zhongshan Hospital of Xiamen University; Heping Hospital affiliated to Changzhi Medical College; Nanjing Drum Tower Hospital; the First School of Clinical Medicine, Lanzhou University; Affiliated Hospital of Xuzhou Medical University; Chifeng Municipal Hospital; Hebei General Hospital; Central People's Hospital of Zhanjiang; Zhumadian Central Hospital; the Second Affiliated Hospital of Harbin Medical University; Liaocheng People's Hospital; Ordos Central Hospital; the First Affiliated Hospital of Shihezi University; Yichun People's Hospital; Longyan First Hospital; the Second People's Hospital Medical Group of Qinzhou; Luohe Central Hospital; Qinghai Provincial People's Hospital; Sichuan Provincial People's Hospital; the People's Hospital of Yuxi City; Jiuquan City People's Hospital; the People's Hospital of Baise; Dalian Municipal Central Hospital; Xingyi People's Hospital; the Affiliated Changsha Central Hospital; 3201 Hospital of Xi'an Jiaotong University Health Science Center; Xiangyang No. 1 People's Hospital; Yan'an University Affiliated Hospital; People's Hospital of Xinjiang Uygur Autonomous Region; Shenzhen Second People's Hospital; the People's Hospital of Xizang Autonomous Region; Shaoxing People's Hospital; Tian Shui First People's Hospital; the Third Affiliated Hospital of Guangzhou Medical University; Meizhou People's Hospital; Anqing Municipal Hospital; Xingtai People's Hospital; First Affiliated Hospital of Anhui University of Science and Technology; the Second Affiliated Hospital of Kunming Medical University; Ankang Central Hospital; Xinyang Central Hospital; Hubei Third People's Hospital; Affiliated Hospital of Jiujiang University; the Second Affiliated Hospital of Hainan Medical University; the First Affiliated Hospital of Jinzhou Medical University; Wuzhou Workers' Hospital; Yueyang Central Hospital; Dali Nationality

Autonomous Prefecture Hospital; Maanshan People's Hospital; Dandong Central Hospital; Chengde Central Hospital; Linfen Central Hospital; Jinzhong First People's Hospital; Tongji University Affiliated Shanghai Fourth People's Hospital; the First Hospital of Nanping City; Daqing Oilfield General Hospital; Jilin Province People's Hospital; the Second People's Hospital of Mudanjiang City; the First Hospital of Qiqihar; Tonghua Central Hospital; Karamay Central Hospital; Shannan People's Hospital of Tibet; Xining No. 1 People's Hospital; Sinopharm Dongfeng General Hospital; the Second Clinical College of North Sichuan Medical College; Shenyang Tenth People's Hospital; Hunan Brain Hospital; the Second Affiliated Hospital of Guizhou Medical University; Sanya People's Hospital; Huzhou Central Hospital; Baiyin First People's Hospital; Xiaogan Central Hospital; Jilin City Central Hospital; Siping City Central Hospital; Wuzhong City People's Hospital; Zigong First People's Hospital; Suining Central Hospital.

## Methods

### Propensity score model for stabilized inverse probability of treatment weighting (IPTW)

We chose stabilized IPTW methods to estimate treatment effects in our primary analyses. IPTW was used to balance baseline characteristics between the 2 groups, which is more similar to the procedure in randomized controlled trials. The stabilization feature of this model ensures the preservation of the study population's size. Patients in the endovascular treatment (EVT) group were weighted (pt/propensity score), patients in the standard care group were weighted [(1 – pt)/(1 – propensity score)], "pt" represents the ratio of the number of individuals in the EVT group to the total population.

### Missing data

The information in the multicenter registry study of cerebral venous thrombosis in China registry is extracted from the hospital inpatient database, providing relatively comprehensive content. Despite this, there are some lost-to-follow-up cases and missing data. The missing data records primarily include baseline modified Rankin Scale (mRS) scores and baseline National Institutes of Health Stroke Scale (NIHSS) scores. The number of lost-to-follow-up cases in adults is 231, accounting for 7.5% of the total population (231/3063). Because the proportion is not particularly high, lost-to-follow-up patients have not been included in the analysis of the effectiveness and safety of adult EVT. In the population with follow-up data, there are 4 cases with missing baseline mRS scores and 57 cases with missing baseline NIHSS scores (**Fig. 1**).

### Sensitivity analysis using propensity score matched cohort

In the sensitivity analyses, we employed an alternative propensity score method, specifically propensity score matching. The propensity score matching was carried out using a 1:1 nearest-neighbor matching algorithm without replacement. A caliper width equal to 0.2 of the standard deviation of the logit of the propensity score was employed.

**Table S1** Characteristics of patients by different levels of hospitals

| Characteristics | EVT | | | Standard care | | |
|---|---|---|---|---|---|---|
| | **Provincial hospital (*n* = 305)** | **Prefecture-level hospital (*n* = 144)** | ***P*-value** | **Provincial hospital (*n* = 1102)** | **Prefecture-level hospital (*n* = 1223)** | ***P*-value** |
| Age (years, mean ± SD) | 37.9 ± 13.2 | 40.3 ± 14.5 | < 0.01 | 41.5 ± 15.0 | 44.3 ± 15.8 | < 0.01 |
| Sex [*n* (%)] | | | < 0.01 | | | 0.89 |
| Male | 160 (52.5) | 65 (45.1) | | 543 (49.3) | 610 (49.9) | |
| Female | 145 (47.5) | 79 (54.9) | | 559 (50.7) | 613 (50.1) | |
| Baseline NIHSS score [*n* (%)] | | | < 0.01 | | | < 0.01 |
| 0 − 1 | 222 (72.8) | 73 (50.7) | | 895 (81.2) | 911 (74.5) | |
| 2 − 4 | 38 (12.5) | 34 (23.6) | | 121 (11.0) | 180 (14.7) | |
| ≥ 5 | 45 (14.8) | 37 (25.7) | | 86 (7.8) | 132 (10.8) | |
| Baseline mRS score [*n* (%)] | | | < 0.01 | | | < 0.01 |
| 0 − 1 | 199 (65.2) | 64 (44.4) | | 803 (72.9) | 794 (64.9) | |
| 2 − 5 | 106 (34.8) | 80 (55.6) | | 299 (27.1) | 429 (35.1) | |

*EVT* endovascular treatment, *NIHSS* National Institutes of Health Stroke Scale, *mRS* modified Rankin Scale

**Table S2** Components of endovascular treatment (EVT) for the patients (%)

| Characteristics | Components of EVT for all hospitals | Components of EVT for provincial hospitals | Components of EVT for prefecture-level hospitals |
|---|---|---|---|
| MT | 30.10 | 24.30 | 42.40 |
| IT | 29.60 | 33.10 | 22.20 |
| IT + MT | 19.80 | 19.60 | 18.80 |
| IS | 6.50 | 8.50 | 2.10 |
| IAT | 4.00 | 3.70 | 4.20 |
| MT + IS | 3.60 | 4.20 | 2.10 |
| IT + MT + IS | 3.30 | 3.20 | 3.50 |
| IT + IS | 1.10 | 1.60 | 0.00 |
| IAT + MT | 0.90 | 0.98 | 1.38 |
| IAT + IT + MT + IS | 0.70 | 0.70 | 2.00 |
| IAT + IT + MT | 0.50 | 0.00 | 1.40 |
| IAT + MT + IS | 0.50 | 0.30 | 0.60 |

*IAT* denotes Intra-arterial thrombolysis, *IT* intrasinus thrombolysis, IS intrasinus stenting, *MT* mechanical thrombectomy

**Table S3** Characteristics of patients, before and after propensity score matching

| Characteristics | All patients | | | Propensity score matched patients | | |
|---|---|---|---|---|---|---|
| | **EVT[a] (*n* = 449)** | **Standard care (*n* = 2325)** | ***P*-value** | **EVT (*n* = 445)** | **Standard care (*n* = 445)** | **SMD[b]** |
| Age (years, mean ± SD) | 38.7 ± 13.7 | 43.0 ± 15.5 | 0.001 | 38.7 ± 13.7 | 38.4 ± 13.7 | 0.054 |
| Female [*n* (%)] | 224 (49.9) | 1172 (50.4) | 0.881 | 223 (50.1) | 226 (50.8) | 0.013 |
| Symptoms [*n* (%)] | | | | | | |
| Vomiting | 200 (44.5) | 857 (36.9) | 0.003 | 247 (55.5) | 253 (56.9) | 0.018 |
| Seizure | 108 (24.1) | 393 (16.9) | 0.001 | 338 (76.0) | 342 (76.9) | 0.087 |
| Consciousness disturbance | 120 (26.7) | 353 (15.2) | 0.001 | 327 (73.5) | 327 (73.5) | 0.073 |
| Motor deficit | 141 (31.4) | 444 (19.1) | 0.001 | 307 (69.0) | 306 (68.8) | 0.015 |
| Sensory deficit | 62 (13.8) | 145 (6.2) | 0.001 | 387 (87.0) | 385 (86.5) | 0.020 |
| Medical history [*n* (%)] | | | | | | |
| Hypertension | 66 (14.7) | 442 (19.0) | 0.036 | 379 (85.2) | 375 (84.3) | 0.031 |
| Diagnosis [*n* (%)] | | | | | | |
| Cerebral hemorrhage | 129 (28.7) | 408 (17.5) | 0.001 | 319 (71.7) | 329 (73.9) | 0.061 |
| Subarachnoid hemorrhage | 58 (12.9) | 159 (6.8) | 0.001 | 389 (87.4) | 394 (88.5) | 0.014 |
| Pulmonary infection | 61 (13.6) | 171 (7.4) | 0.001 | 387 (87.0) | 385 (86.5) | 0.048 |
| Drug therapy in hospital [*n* (%)] | | | | | | |
| Warfarin | 215 (47.9) | 780 (33.6) | 0.001 | 232 (52.1) | 232 (52.1) | 0.001 |
| Rivaroxaban | 43 (9.6) | 374 (16.1) | 0.001 | 402 (90.3) | 409 (91.9) | 0.064 |

| Characteristics | All patients | | | Propensity score matched patients | | |
|---|---|---|---|---|---|---|
| | **EVT[a] (*n* = 449)** | **Standard care (*n* = 2325)** | ***P*-value** | **EVT (*n* = 445)** | **Standard care (*n* = 445)** | **SMD[b]** |
| Baseline NIHSS score [*n* (%)] | | | 0.001 | | | 0.077 |
| 0 −1 | 295 (65.7) | 1806 (77.7) | | 293 (65.8) | 286 (64.3) | |
| 2 −4 | 72 (16.0) | 301 (12.9) | | 72 (16.2) | 74 (16.6) | |
| ≥ 5 | 82 (18.3) | 218 (9.4) | | 80 (18.0) | 85 (19.1) | |
| Baseline mRS score[c] [*n* (%)] | | | | | | |
| 2 −5 | 186 (41.4) | 728 (31.3) | 0.001 | 183 (41.1) | 194 (43.6) | 0.018 |
| Hospital level [*n* (%)] | | | | | | |
| Prefecture-level hospital | 144 (32.1) | 1223 (52.6) | 0.001 | 144 (32.4) | 149 (33.5) | 0.005 |
| Follow-up time[d] [days, mean ± SD] | 822.3 ± 492.4 | 902.4 ± 507.3 | 0.002 | 824.9 ± 491.8 | 831.4 ± 531.4 | 0.044 |

[a]Endovascular treatment (EVT) includes intra-arterial thrombolysis, intrasinus thrombolysis, intrasinus stenting, and mechanical thrombectomy. [b]The difference between the groups divided by SMD; a value greater than 10% is interpreted as a meaningful difference. [c]Scores on the mRS of functional recovery range from 0 to 6, with higher scores indicating more severe disability and 6 indicating death. [d]Follow-up time refers to the duration from the patient's discharge to the current point of follow-up. *SMD* standardized mean difference, *NIHSS* National Institutes of Health Stroke Scale, *mRS* modified Rankin Scale

**Table S4** Sensitivity analyses of primary, secondary outcomes, and safety outcomes using propensity score matching

| Outcome | EVT[a] [*n* = 449, *n* (%)] | Standard care [*n* = 2325, *n* (%)] | Unmatched patients | | Propensity score matched patients |
|---|---|---|---|---|---|
| | | | Unadjusted risk ratio (95% CI) | Adjusted risk ratio (95% CI)[b] | Matched risk ratio (95% CI)[c] |
| Primary outcome | | | | | |
| mRS score at present[d] | | | | | |
| 0 − 1 | 379 (84.4) | 2102 (90.4) | 0.93 (0.90 − 0.97) | 0.98 (0.95 − 1.02) | 0.97 (0.92 − 1.03) |
| Secondary outcomes | | | | | |
| mRS score at present | | | | | |
| 0 − 2 | 404 (90.0) | 2178 (93.7) | 0.96 (0.93 − 0.99) | 1.00 (0.97 − 1.03) | 0.99 (0.95 − 1.04) |
| 0 − 3 | 414 (92.2) | 2203 (94.8) | 0.97 (0.95 − 1.00) | 1.01 (0.98 − 1.03) | 1.00 (0.96 − 1.03) |
| mRS score at discharge | | | | | |
| 0 − 1 | 300 (66.8) | 1848 (79.5) | 0.84 (0.79 − 0.89) | 0.94 (0.89 − 1.00) | 0.89 (0.82 − 0.97) |
| 0 − 2 | 349 (77.7) | 2063 (88.9) | 0.88 (0.84 − 0.91) | 0.96 (0.92 − 1.00) | 0.93 (0.88 − 1.00) |
| 0 − 3 | 380 (84.6) | 2148 (92.4) | 0.92 (0.89 − 0.95) | 0.99 (0.96 − 1.03) | 0.98 (0.93 − 1.03) |
| Safety outcomes | | | | | |
| Death at discharge | 18 (4.0) | 24 (1.0) | 3.88 (2.09 − 7.09) | 1.59 (0.64 − 3.25) | 1.89 (0.87 − 4.38) |
| Death at present | 23 (5.1) | 77 (3.3) | 1.55 (0.96 − 2.40) | 1.02 (0.63 − 1.61) | 0.96 (0.54 − 1.70) |

[a]Endovascular treatment (EVT) includes intra-arterial thrombolysis, intrasinus thrombolysis, intrasinus stenting, and mechanical thrombectomy. [b]Adjustments were made for age, sex (male or female), symptoms (vomiting, seizure, consciousness disturbance, coma, motor deficit, sensory deficit, hypertension), diagnosis (cerebral hemorrhage, subarachnoid hemorrhage, pulmonary infection), drug therapy in hospital (warfarin, rivaroxaban), baseline NIHSS score (0 − 1, 2 − 4 or ≥ 5), baseline mRS score (0 − 1or 2 − 5), and follow-up time. [c]Shown is the risk ratio from a modified Poisson regression model and covariates with inverse probability weighting according to the propensity score. [d]Scores on the mRS of functional recovery range from 0 to 6, with higher scores indicating more severe disability and 6 indicating death. *CI* confidence interval, *NIHSS* National Institutes of Health Stroke Scale, *mRS* modified Rankin Scale

**Table S5** Different endovascular treatment (EVT) and outcomes among patients with cerebral venous thrombosisa (CVT)[a]

| **Outcome** | **MT [*n* = 135, *n* (%)]** | **Other EVT [*n* = 314, *n* (%)]** | **Unweighted patients** | | **Propensity score weighted patients** |
|---|---|---|---|---|---|
| | | | **Unadjusted risk ratio (95% CI)** | **Adjusted risk ratio (95% CI)[b]** | **Weighted risk ratio (95% CI)[c]** |
| Primary outcome | | | | | |
| mRS score at present[d] | | | | | |
| 0 – 1 | 111 (82.2) | 268 (85.3) | 0.79 (0.46 – 1.36) | 0.93 (0.45 – 1.92) | 0.79 (0.46 – 1.37) |
| 2 – 6 | 24 (17.8) | 46 (14.7) | 1.00 | 1.00 | 1.00 |
| Secondary outcomes | | | | | |
| mRS score at present | | | | | |
| 0 – 2 | 115 (85.2) | 289 (92.0) | 0.50 (0.27 – 0.93) | 0.45 (0.19 – 1.08) | 0.50 (0.27 – 0.93) |
| 3 – 6 | 20 (14.8) | 25 (8.0) | 1.00 | 1.00 | 1.00 |
| mRS score at present | | | | | |
| 0 – 3 | 122 (90.3) | 292 (93.0) | 0.71 (0.35 – 1.45) | 0.91 (0.33 – 2.52) | 0.71 (0.34 – 1.45) |
| 4 – 6 | 13 (9.7) | 22 (7.0) | 1.00 | 1.00 | 1.00 |
| mRS score at discharge | | | | | |
| 0 – 1 | 88 (65.2) | 212 (67.5) | 0.90 (0.59 – 1.38) | 1.10 (0.57 – 2.13) | 0.90 (0.59 – 1.38) |
| 2 – 6 | 47 (34.8) | 102 (32.5) | 1.00 | 1.00 | 1.00 |
| mRS score at discharge | | | | | |
| 0 – 2 | 106 (78.5) | 243 (77.4) | 1.07 (0.66 – 1.74) | 1.61 (0.76 – 3.40) | 1.07 0.65 – 1.74) |
| 3 – 6 | 29 (21.5) | 71 (22.6) | 1.00 | 1.00 | 1.00 |
| mRS score at discharge | | | | | |
| 0 – 3 | 111 (82.2) | 269 (85.7) | 0.77 (0.45 – 1.33) | 0.96 (0.41 – 2.23) | 0.77 (0.45 – 1.33) |
| 4 – 6 | 24 (17.3) | 45 (14.3) | 1.00 | 1.00 | 1.00 |

| Outcome | MT [*n* = 135, *n* (%)] | Other EVT [*n* = 314, *n* (%)] | Unweighted patients | | Propensity score weighted patients |
|---|---|---|---|---|---|
| | | | Unadjusted risk ratio (95% CI) | Adjusted risk ratio (95% CI)[b] | Weighted risk ratio (95% CI)[c] |
| Safety outcomes | | | | | |
| Death at discharge | 8 (6.0) | 10 (3.0) | 1.91 (0.74 − 4.96) | 1.29 (0.24 − 7.09) | 1.91 (0.74 − 4.97) |
| Survival at discharge | 127 (94.0) | 304 (97.0) | 1.00 | 1.00 | 1.00 |
| Safety outcomes | | | | | |
| Death at present | 10 (7.0) | 13 (4.0) | 1.85 (0.79 − 4.34) | 1.05 (0.37 − 3.03) | 1.85 (0.79 − 4.34) |
| Survival at present | 125 (93.0) | 301 (96.0) | 1.00 | 1.00 | 1.00 |

[a]EVT includes use of intra-arterial thrombolysis, intrasinus thrombolysis, intrasinus stenting, and mechanical thrombectomy (MT); MT denotes separate mechanical thrombectomy; other EVT denotes EVT treatments other than standalone MT; confidence intervals for secondary outcomes were not adjusted for multiple comparisons, and no clinical inferences can be made from differences between groups. [b]Adjustments were made for age, sex (male or female), symptoms (vomiting, seizure, consciousness disturbance, motor deficit, sensory deficit, hypertension), diagnosis (cerebral hemorrhage, subarachnoid hemorrhage, pulmonary infection), drug therapy in hospital (warfarin, rivaroxaban), baseline NIHSS score (0 − 1, 2 − 4 or ≥ 5), baseline mRS score (0 – 1 or 2 − 5), and follow-up time. [c]Shown is the risk ratio from a modified Poisson regression model and covariates with inverse probability weighting according to the propensity score. [d]Scores on the mRS of functional recovery range from 0 to 6, with higher scores indicating more severe disability and 6 indicating death. *CI* confidence interval, *NIHSS* National Institutes of Health Stroke Scale, *mRS* modified Rankin Scale

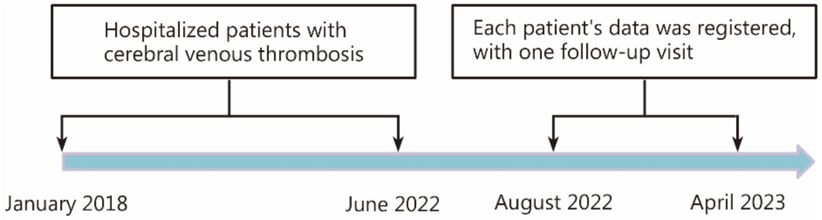


**Fig. S1** Study flow chart timeline

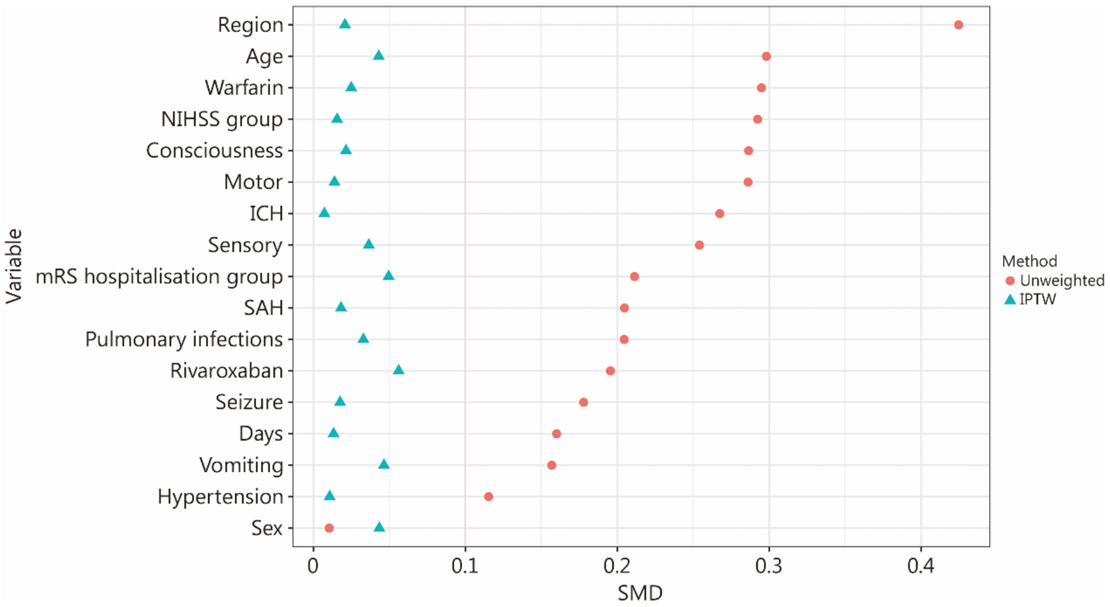


**Fig. S2** Standardized mean differences in the unweighted and weighted patients. Days indicate the time from hospital discharge to the date of assessment. IPTW inverse probability of treatment weighting, ICH intracranial hemorrhage, SAH subarachnoid hemorrhage, SMD standardized mean differences, NIHSS National Institutes of Health Stroke Scale, mRS modified Rankin Scale

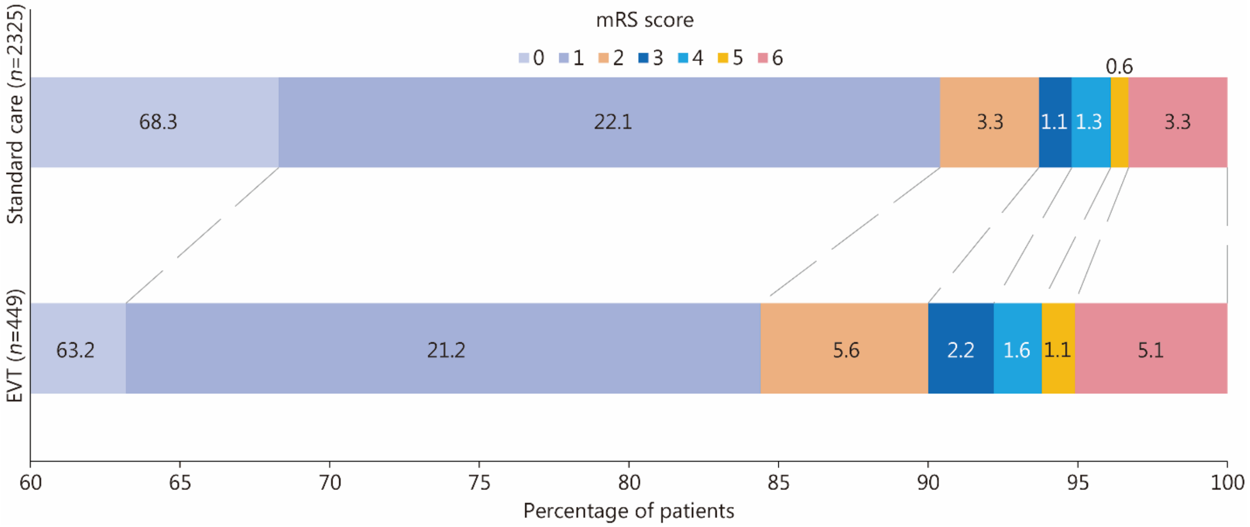


**Fig. S3** Distribution of the modified Rankin Scale (mRS) score at present in all patients. Scores on the mRS range from 0 to 6, with a score of 0 indicating no symptoms, 1 no clinically significant disability, 2 slight disability (able to look after their own affairs without assistance but unable to carry out all previous activities), 3 moderate disability ( require some help but ability to walk unassisted), 4 moderately severe disability (unable to attend to bodily needs and unable to walk unassisted), 5 severe disability (require constant nursing care and attention), and 6 death. EVT endovascular treatment
